# Clinical Implications of Postoperative Hyperamylasemia after Partial Pancreaticoduodenectomy

**DOI:** 10.1159/000526495

**Published:** 2022-10-24

**Authors:** Ioannis Mintziras, Sabine Wächter, Jerena Manoharan, Veit Kanngiesser, Elisabeth Maurer, Detlef K. Bartsch

**Affiliations:** Department of Visceral-, Thoracic- and Vascular Surgery, Philipps-University Marburg, Marburg, Germany

**Keywords:** Postoperative hyperamylasemia, Postoperative acute pancreatitis, Postoperative pancreatic fistula, Partial pancreaticoduodenectomy

## Abstract

**Introduction:**

The present study aimed to examine the clinical implications of postoperative hyperamylasemia (POH) after partial pancreaticoduodenectomy (PD).

**Methods:**

Data from all consecutive patients undergoing PD were obtained from a prospectively maintained database and reviewed. POH was defined as an elevation of serum pancreatic amylase above the upper limit of normal (53 U/L) on postoperative days 0–2. Clinically relevant POH (cr-POH) was defined as POH in patients with clinically relevant (Clavien-Dindo ≥ III) postoperative complications.

**Results:**

POH occurred in 61 of 170 (35.9%) and cr-POH in 24 of 170 (14.1%) patients. Patients with POH had higher rates of clinically relevant postoperative pancreatic fistula (cr-POPF) (44.3 vs. 3.7%, *p* < 0.001) and clinically relevant postoperative complications than those without POH (39.3 vs. 21.1%, *p* = 0.001). Patients with cr-POH had higher C-reactive protein (CRP, milligrams per liter) levels on third (257.7 vs. 187.85 mg/L, *p* = 0.016) and fourth (222.5 vs. 151, *p* = 0.002) postoperative day (POD) than those with POH alone. Serum procalcitonin (PCT, micrograms per liter) levels on POD 2 (1.2 vs. 0.4 μg/L, *p* = 0.028) and POD 3 (0.85 vs. 0.4 μg/L, *p* = 0.001) were also higher in patients with cr-POH. Rates of cr-POPF in patients with cr-POH were higher than in those with POH alone (70.8 vs. 27%, *p* = 0.001). POH (OR 0.011, 95% CI: 0.001–0.097, *p* < 0.001) was an independent predictor of cr-POPF in the multivariable analysis. A high-risk pathology, defined as nonadenocarcinoma/nonchronic pancreatitis pathology (OR 0.277, 95% CI: 0.106–0.727, *p* = 0.009), and a small duct diameter (OR 0.333, 95% CI: 0.139–0.796, *p* = 0.013) were independent predictors of POH in the multivariable analysis.

**Conclusion:**

POH is a frequent, but not always clinically relevant, finding after partial PD. Serum CRP and PCT levels in the early postoperative period can be used to identify patients with cr-POH. POH is an independent risk factor for increased postoperative morbidity, including cr-POPF, after partial PD.

## Introduction

Pancreatic cancer is the fourth leading cause of cancer-related mortality in the Western world and predicted to be the second leading cause of cancer death by the year 2030 [1, 2]. Although postoperative mortality after partial pancreaticoduodenectomy (PD) has been reduced to rates below 5% in specialized centers [3–5], postoperative morbidity has not been significantly reduced in the past decades, occurring in up to 40–50% of patients [3, 5–7]. Several studies assessing different surgical techniques for PD have failed to identify technical aspects as risk factors for the development of clinically relevant postoperative pancreatic fistula (cr-POPF) [8–11]. A recent systematic review examining POPF-related mortality rates in 60,739 patients showed that Grade C POPFs were associated with a postoperative mortality rate of 25.7% [12]. Therefore, POPF remains the main source of major morbidity and mortality after PD, with a reported incidence of up to 35% [5, 7, 13, 14]. This is possibly due to the fact that the underlying mechanism of POPF is poorly understood [15]. The role of postoperative acute pancreatitis (POAP) in the development of POPF has been the subject of increasing investigation in recent time [16–20]. POAP was first defined by Connor as the elevation of the serum amylase level above the upper normal limit on postoperative days (POD) 0–2 [21]. Studies investigating POAP according to the definition proposed by Connor identified a significant association between an increase in serum amylase and postoperative morbidity [16–20, 22]. However, more recent studies [23] showed that postoperative hyperamylasemia (POH) and POAP are two different entities and that the diagnosis of POAP cannot be based on biochemical evidence only [23]. In this direction, the newly proposed definition of the International Study Group of Pancreatic Surgery distinguishes between POH as a mere biochemical entity and postpancreatectomy pancreatitis, in patients with radiological and clinical criteria of postoperative pancreatitis [24]. Since POH occurs early in the postoperative period, it could be used as a predictive factor for severe postoperative morbidity after partial PD. Therefore, the aim of the present study was to examine the clinical impact of POH on the occurrence of clinically relevant postoperative complications, including cr-POPF, as well as to identify risk factors for POH after partial PD.

## Materials and Methods

### Data Collection

Data from all consecutive patients who underwent an elective, open partial PD between January 2009 and December 2019 in University Hospital Marburg were obtained from a prospectively maintained database and retrospectively reviewed. The results of the present study are reported in line with the STROBE guidelines [25]. Preoperative clinical data included age, sex, body mass index (BMI), the American Society of Anaesthesiologists (ASA) status, preoperative biliary drainage, serum albumin (grams per deciLiter), preoperative serum lipase (units per liter), and/or serum amylase (units per liter). Patients were excluded from the present study if preoperative serum amylase and lipase were not documented. Intraoperative data included assessment of the main pancreatic duct size (millimeters) and pancreatic gland texture (soft/firm), estimated blood loss (milliliters), blood transfusion, surgical procedure performed, simultaneous vascular resection, and operative time (minutes).

### Surgical Technique and Perioperative Management

All procedures were done by laparotomy. All patients received a single-shot intravenous antibiotic, usually ampicillin/sulbactam 30 min prior to laparotomy. Somatostatin analogues were also routinely used perioperatively. A pylorus-preserving pancreaticoduodenectomy with reconstruction through a pancreatogastrostomy was the procedure of choice, when feasible. Otherwise, a standard Whipple procedure was performed. Pancreatic duct stents were routinely used, and three “easy flow” drains (LightFlow, Ruesch, 12 mm) were also placed adjacent to the anastomosis. The rightsided paired drains were placed posterior to the hepaticojejunostomy and caudal to the pancreatogastrostomy. The left-sided drain was placed cranial to the pancreatogastrostomy. Amylase values in drains were controlled on POD 1, 3, and 5, and the drains were removed on POD 5 in patients with an uneventful clinical course and no biochemical signs of POPF.

### Definition of Outcomes

Postoperative outcomes, including pancreatic fistula, clinically relevant complications, length of hospital stay, and postoperative mortality, were assessed. POPF was classified according to the most recent definition provided by the International Study Group of Pancreatic Surgery (ISGPS) [26], and grade B and C POPF was defined as cr-POPF. Delayed gastric emptying (DGE) as well as postpancreatectomy hemorrhage (PPH) were defined according to the proposed definitions of the ISGPS [27, 28]. Postoperative complications were classified according to the Clavien-Dindo classification system [29]. POH was defined as an elevation of serum pancreatic amylase levels above the upper limit of normal (53 U/L) on POD 0–2. Clinically relevant POH (cr-POH) was defined as POH combined with clinically relevant (Clavien-Dindo ≥ III) complications. The length of hospital stay was defined as the number of days from surgery to the date of discharge. Postoperative mortality included deaths occurring prior to hospital discharge or within 30 days of surgery. High-risk pathology was defined as nonadenocarcinoma and nonchronic pancreatitis pathology, for example, pancreatic neuroendocrine neoplasia, cystic lesion, and intraductal papillary mucinous neoplasm.

### Statistical Analysis

Continuous variables are expressed as medians with interquartile ranges (IQR), and categorical variables are presented as proportions. Quantitative variables were compared using the Student's *t* test or Mann-Whitney U test and qualitative variables using the χ^2^ test or Fisher's exact test as appropriate. Factors significantly associated on univariate analysis were included in a multivariable binary logistic regression model. All reported probability values (*p* values) are based on two-sided tests, and the level of statistical significance was set at *p* < 0.05. Analyses were performed using SPSS 23.0 (IBM Corp. Released 2015. IBM SPSS Statistics for Windows, Version 23.0; IBM Corp., Armonk, NY, USA).

## Results

### Patients’ Characteristics and Postoperative Outcomes

198 consecutive patients who underwent an elective PD were considered for inclusion. Sixteen patients (8.1%) with a preoperative elevated serum amylase/lipase and 12 patients (6.1%) with incomplete information were excluded from the study cohort. Thus, 170 patients were included in the present study. Ninety-one (53.5%) patients were male, the median age at surgery was 67 (IQR, 56–75), and the median patient BMI was 24.7 kg/m^2^ (IQR, 22.6–28.3). Eighty-eight (51.8%) patients were ASA II, 77 (45.3%) ASA III, 3 (1.8%) ASA I, and 2 (1.2%) patients ASA IV. The general and perioperative characteristics of the study population are summarized in Table 1. Median operative time was 362.5 min (IQR, 315–416.25), median estimated blood loss was 300 mL (IQR, 200–400), and intraoperative blood transfusion was necessary in 9 of 170 (5.3%) patients. A pylorus-preserving pancreaticoduodenectomy was performed in 145 (85.3%) and a standard Whipple procedure in the remaining 25 (14.7%) patients. A vascular resection, for example, portal vein or superior mesenteric vein resection, was performed in 15 (8.8%) patients. A soft pancreatic gland texture was reported in 44 (25.9%) patients, the median estimated pancreatic duct diameter was 3 mm (IQR, 2–6). The most frequent underlying pathology was pancreatic ductal adenocarcinoma in 84 (49.4%) patients, followed by ampullary carcinoma and pancreatic neuroendocrine neoplasia in 16 (9.4%) patients each. Other pathologies included distal bile duct carcinoma in 13 (7.6%) patients, intraductal papillary mucinous neoplasm in 12 (7.1%) patients, chronic pancreatitis in 8 (4.7%) patients, duodenal carcinoma in 4 (2.4%) patients, and various pathologies in the remaining 17 (10%) patients. The median tumor size was 2.7 cm (IQR, 2.3–3.5) and the median number of resected lymph nodes was 21 (IQR, 17–26). In patients with malignant disease, an R0 resection was reported in 116 of 133 (87.2%) patients.

Clinically relevant postoperative complications (Clavien-Dindo ≥ III) occurred in 47 (27.6%) patients that were classified grade IIIa in 17 (10%) patients, grade IIIb in 18 (10.6%), grade IVa in 3 (1.8%), grade IVb in 2 (1.2%), and grade V in 7 (4.1%) patients. A cr-POPF was reported in 31 (18.2%) patients, including 21 (12.4%) patients with grade B and 10 (5.9%) patients with grade C POPF. Grade B POPF was managed by persistent drainage in 14 patients and percutaneous abscess drainage in 7 patients. Of the 10 patients with grade C POPF, 7 patients underwent completion pancreatectomy with splenectomy, in one case the anastomotic leakage was sutured, and the remaining 2 patients with grade C POPF died due to septic shock prior to reoperation. Among the patients undergoing completion pancreatectomy, the histological examination showed signs of acute, severe, necrotizing pancreatitis in 5 of 7 patients. Clinically relevant DGE grade B and C was reported in 38 (22.4%) and 24 (14.1%) patients, respectively. A clinically relevant PPH (Grade B/C) was reported in 21 (12.4%) patients. The median length of hospital stay was 15 days (IQR, 13–20).

### Incidence and Clinical Relevance of POH after Partial PD

POH occurred in 61 of 170 (35.9%) and cr-POH in 24 of 170 (14.1%) patients. In patients without POH, clinically relevant postoperative complications (Clavien-Dindo ≥ III) were reported in 23 of 109 (21.1%) patients, including Grade IIIa in 11 (10.1%), IIIb in 10 (9.2%), and V in 2 (1.8%) patients. Four of 109 (3.7%) patients without POH developed cr-POPF, including 3 Grade B (2.8%) and 1 Grade C (0.9%) POPF. Twenty four of 61 (39.3%) patients with POH developed clinically relevant postoperative complications, including Grade IIIa in 6 (9.8%), IIIb in 8 (13.1%), IVa in 3 (4.9%), IVb in 2 (3.3%), and V in 5 (8.2%) patients. In patients with POH, cr-POPF was reported in 27 of 61 (44.3%) patients, including Grade B in 18 (29.5%) and Grade C in 9 (14.8%) patients. Patients developing POH had significantly higher rates of cr-POPF (44.3 vs. 3.7%, *p* < 0.001) and clinically relevant postoperative complications (39.3 vs. 21.1%, *p* = 0.001). Length of hospital stay was also significantly longer in patients with POH compared to those without POH (17 vs. 14 days, *p* = 0.005). PPH was not significantly different in between groups (16.4 vs. 10.1%, *p* = 0.26). There was a trend toward statistical significance regarding the rates of DGE in favor of the patient group without POH (70.5 vs. 57.8%, *p* = 0.08).

### Subgroup Analysis of Patients with Postoperative Hyperamylasemia

Among the 61 patients with POH, clinically relevant (Clavien-Dindo ≥ III) postoperative complications occurred in 24 of 61 (39.3%) patients with POH, who therefore consisted of the subgroup of patients with cr-POH.

Patients with cr-POH had significantly higher serum C-reactive protein (CRP) (milligrams per liter) levels on third (257.7 vs. 187.85 mg/L, *p* = 0.016) and fourth (222.5 vs. 151 mg/L, *p* = 0.002) POD than those with POH. In addition, serum procalcitonin (PCT) (micrograms per liter) levels on POD 2 (1.2 vs. 0.4 μg/L, *p* = 0.028) and 3 (0.85 vs. 0.4 μg/L, *p* = 0.001) were significantly higher in patients with cr-POH.

Rates of cr-POPF in patients with cr-POH were higher than in those with POH (70.8 vs. 27%, *p* = 0.001). DGE (grade B/C) was reported in 7 of 37 (18.9%) patients with POH versus 16 of 24 (66.7%) patients with cr-POH (*p* < 0.001). Length of hospital stay was significantly longer in patients with cr-POH compared to those with POH (24 vs. 14 days, *p* = 0.001).

### Predictive Factors for Cr-POPF

A soft pancreatic texture (OR 0.370, 95% CI: 0.144–0.955, *p* = 0.04), a small (≤3 mm) duct diameter (OR 0.360, 95% CI: 0.160–0.810, *p* = 0.014), and POH (OR 0.048, 95% CI: 0.016–0.147, *p* < 0.001) were significant predictors of cr-POPF in the univariate analysis, as shown in Table 2. POH (OR 0.011, 95% CI: 0.001–0.097, *p* < 0.001) remained the only independent predictor in the multivariable analysis (Table 2).

### Predictive Factors for POH

Table 3 reports the univariate and multivariable analyses of possible predictive factors of POH. A duct diameter ≤3 mm (OR 0.248, 95% CI: 0.128–0.482, *p* < 0.001), soft pancreatic texture (OR 0.257, 95% CI: 0.119–0.557, *p* = 0.001), and high-risk pathology (OR 0.154, 95% CI: 0.077–0.311, *p* < 0.001) were significant predictors for POH in the univariate analysis. There was also a trend toward statistical significance regarding longer operating time (OR 1.763, 95% CI: 0.935–3.326, *p* = 0.08), simultaneous vascular resection (OR 3.995, 95% CI: 0.871–18.331, *p* = 0.075), and BMI >24.7 kg/m^2^ (OR 1.830, 95% CI: 0.970–3.454, *p* = 0.062). A high-risk pathology (OR 0.277, 95% CI: 0.106–0.727, *p* = 0.009) as well as a small (≤3 mm) duct diameter (OR 0.333, 95% CI: 0.139–0.796, *p* = 0.013) remained independent predictors of POH in the multivariable analysis, as shown in Table 3.

## Discussion

POPF remains the main source of major morbidity and mortality after PD. The identification of risk factors and also objective ways to accurately predict the occurrence of POPF and especially cr-POPF (grade B and C according to ISGPS [26]) has been and continue to be the subject of many clinical studies. Along with soft pancreatic texture and small duct diameter [5, 30–32], biochemical markers including amylase value in drains [33–35] and serum CRP [31, 35, 36] have proven to be reliable predictors for the occurrence of POPF in several studies. Moreover, POH has been suggested as a potential biochemical marker of the intraoperative pancreatic trauma and the resulting POAP associated with the occurrence of cr-POPF. Connor [21] suggested that the intraoperative transient hypoperfusion can induce pancreatic necrosis and lead to pancreatic anastomotic failure. However, Connor's definition of POAP was merely based on the biochemical evidence of pancreatic inflammation, namely serum amylase activity greater than the upper limit of normal between POD 0–2 [21], which led to a reported incidence of POAP as high as 64% [37], as shown in a recent systematic review. In many cases though, serum amylase was greater on the first POD and decreased with time postoperatively and was often not measured after POD 4 [37], suggesting that the definition of POAP cannot be based on biochemical evidence only. Loos et al. [23] showed that biochemical evidence of hyperamylasemia POD1 is not synonymous with POAP because 58% of patients with hyperamylasemia on POD1 did not have POAP based on radiologic criteria [23]. POH and POAP appear to be two different entities, as suggested in the newly proposed POAP definition proposed by the ISGPS [24]. According to the ISGPS, to be defined as POAP, POH needs to be confirmed by cross-sectional imaging and/or be clinically relevant to the patient [24]. A POH that does not affect the patient's clinical course should not be considered as POAP [23, 24]. Similarly, the present study showed that POH is a frequent finding after partial PD with a reported frequency of 35.9%, results in line with those of previous studies reporting POH rates between 41% and 63.4% [16, 19, 20]. In addition, the term cr-POH was introduced to distinguish patients developing clinically relevant postoperative complications due to POH from those with biochemical evidence only. In the present study, POH was clinically relevant in only 14.1% of patients, suggesting that POH alone is not synonymous to POAP, as shown in the study of Loos et al. [23].

Patients developing POH in the present study had significantly higher rates of cr-POPF (44.3 vs. 3.7%) and clinically relevant postoperative complications (39.3 vs. 21.1%) than those without POH, results in line with those of previous studies [16, 17, 19, 20, 22, 37]. In addition, the present study showed that patients with cr-POH had even higher rates of cr-POPF than those with POH alone (70.8 vs. 27%). Serum CRP levels on POD 3 (257.7 vs. 187.85 mg/L) and POD 4 (222.5 vs. 151 mg/L) as well as serum PCT levels on POD 2 (1.2 vs. 0.4 μg/L) and POD 3 (0.85 vs. 0.4 μg/L) were also significantly higher in patients cr-POH than in those with POH alone, suggesting that these markers could help distinguish POH from cr-POH and identify patients at high risk for developing clinically relevant complications in the early postoperative period.

Moreover, in the present study a soft pancreatic texture, a small (<3 mm) duct diameter, and POH were identified as significant predictors of cr-POPF in the univariate analysis, whereas POH remained the only independent predictor in the multivariable analysis. Similarly, Partelli et al. [20] showed that a soft pancreatic texture, intraoperative blood transfusion, and POAP defined according to Connor [21] were independent predictive factors of cr-POPF in the multivariable analysis. Chen et al. [19] identified several independent predictors of cr-POPF, including POH, a BMI >24 kg/m^2^, small (≤3 mm) duct diameter, intraoperative blood transfusion, and high-risk pathology (pathology other than adenocarcinoma and chronic pancreatitis). Contrary to most previous studies [17, 18, 20, 22], the present study also aimed to identify predictors of POH. A soft pancreatic texture, a small (<3 mm) duct diameter, and a high-risk pathology defined as nonadenocarcinoma and nonchronic pancreatitis pathology were significant predictors of POH in the univariate analysis, whereas high-risk pathology and small duct diameter remained independent predictors in the multivariable analysis. To date, only two studies [16, 19] have examined potential predictive factors of POH after partial PD. A small (≤3 mm) duct diameter was identified as an independent predictor of POH in both previous studies [16, 19]. Chen et al. [19] also reported high-risk pathology as an independent predictive factor of POH, which results in line with those of the present study. Bannone et al. [16] showed that exocrine insufficiency, neoadjuvant therapy, and a soft pancreatic texture along with a small duct diameter could independently predict the occurrence the POH after partial PD.

The retrospective nature of the present study should be acknowledged as its main limitation. Moreover, due to the restrictions in the information held in the prospectively maintained database, some low-grade complications (Clavien-Dindo < III) may have been missed.

In conclusion, POH, defined by the elevation of serum amylase level above the upper normal limit on POD 0–2, is a frequent, but not always clinically relevant, finding after partial PD. In the present study, POH independently predicted cr-POPF and was significantly associated with higher rates of clinically relevant postoperative complications. Prospective studies are needed in order to further examine the association of POH and cr-POH with the occurrence of cr-POPF after partial PD and the role of serum CRP and PCT levels as indicators of cr-POH early in the postoperative period.

## Statement of Ethics

According to the Institutional Review Board of University Hospital Marburg, an IRB approval was not mandatory for conducting the present retrospective analysis. The IRB also waived the need for written informed consent.

## Conflict of Interest Statement

The authors have no conflicts of interest to declare.

## Funding Sources

None to declare.

## Author Contributions

Study conception and design: Ioannis Mintziras and Detlef K. Bartsch. Acquisition, analysis, and data interpretation: Ioannis Mintziras, Jerena Manoharan, and Sabine Wächter. Drafting, revising, and final approval of the manuscript: Ioannis Mintziras, Sabine Wächter, Jerena Manoharan, Elisabeth Maurer, Veit Kanngiesser, and Detlef K. Bartsch.

## Data Availability Statement

All data generated or analyzed during this study are included in this article. Further enquiries can be directed to the corresponding author.

## Figures and Tables

**Table 1:** General and perioperative characteristics of the included patients (*n* = 170)

Variable	Median (IQR) or *n* (%)
Age, years	67 (56–75)
Sex (male)	91 (53.5)
BMI, kg/m^2^	24.7 (22.6–28.3)
ASA I	3 (1.8)
ASA II	88 (51.8)
ASA III	77 (45.3)
ASA IV	2 (1.2)
Operative time, min	362.5 (315–416.25)
Estimated blood loss, mL	300 (200–400)
PPPD	145 (85.3)
Kausch-Whipple	25 (14.7)
Vascular resection	15 (8.8)
Pancreatic duct diameter, mm	3 (2–6)
Pancreatic texture (soft)	44 (25.9)
Underlying pathology	
PDAC	84 (49.4)
Ampullary carcinoma	16 (9.4)
pNEN	16 (9.4)
Distal bile duct carcinoma	13 (7.6)
IPMN	12 (7.1)
Chronic pancreatitis	8 (4.7)
Duodenal carcinoma	4 (2.4)
Various	17 (10)
Tumor size, cm	2.7 (2.3–3.5)
Lymph nodes resected	21 (17–26)
R0 resection^1^	116 of 133 (87.2)
Clavien-Dindo ≥ III (all)	47 (27.6)
Grade IIIa	17 (10)
Grade IIIb	18 (10.6)
Grade IVa	3 (1.8)
Grade IVb	2 (1.2)
Grade V	7 (4.1)
Fistula risk score^2^	
0 (negligible risk)	46 (27.1)
1–2 (low risk)	47 (27.6)
3–6 (intermediate risk)	73 (42.9)
7–10 (high risk)	4 (2.4)
POPF	
Biochemical leak	24 (14.1)
Grade B	21 (12.4)
Grade C	10 (5.9)
LOS	15 (13–20)

Values are median unless indicated otherwise. BMI, body mass index; ASA, American Society Anesthesiology; PPPD, pylorus-preserving pancreaticoduodenectomy; PDAC, pancreatic ductal adenocarcinoma; pNEN, pancreatic neuroendocrine neoplasia; IPMN, intraductal papillary-mucinous neoplasia; LOS, length of hospital stay.

1 Refers to patients with malignant disease.

2 Callery et al. [30].

**Table 2:** Predictors of cr-POPF in the univariate and multivariable analysis

Variable	Univariate analysis			Multivariable analysis
	OR	95% CI	*p* value	OR	95% CI	*p* value
Age, years						
≤67	1					
>67	1.114	0.511–2.427	0.786			
Sex						
Female	1					
Male	0.777	0.356–1.695	0.526			
ASA						
≤II	1					
>II	1.099	0.504–2.395	0.813			
BMI, kg/m^2^						
≤24.7	1					
>24.7	1.803	0.813–3.995	0.147			
Operative time, min						
≤362.5	1					
>362.5	1.082	0.497–2.358	0.843			
Blood loss, mL						
≤300	1					
>300	1.077	0.476–2.433	0.859			
Surgical procedure						
PPPD	1					
Whipple	1.961	0.738–5.207	0.177			
Pancreatic texture						
Soft	1					
Firm	0.370	0.144–0.955	**0.04**	0.857	0.245–3.001	0.810
Duct diameter, mm						
≤3	1					
>3	0.360	0.160–0.810	**0.014**	0.686	0.183–2.568	0.576
POH						
Yes	1					
No	0.048	0.016–0.147	**<0.001**	0.011	0.001–0.097	**<0.001**

cr-POPF, clinically relevant postoperative pancreatic fistula; BMI, body mass index; ASA, American society anesthesiology; PPPD, pylorus-preserving pancreaticoduodenectomy; POH, postoperative hyperamylasemia; OR, odds ratio; 95% CI, 95% confidence interval.

**Table 3:** Predictors of POH in the univariate and multivariable analysis

Variable	Univariate analysis		Multivariable analysis	
	OR	95% CI	*p* value	OR	95% CI	*p* value
Age, years						
≤67	1					
>67	1.486	0.791–2.791	0.218			
Sex						
Female	1					
Male	0.762	0.406–1.428	0.395			
ASA						
≤II	1					
>II	1.274	0.678–2.395	0.452			
BMI, kg/m^2^						
≤24.7	1					
>24.7	1.830	0.970–3.454	0.062			
Operative time, min						
≤362.5	1					
>362.5	1.763	0.935–3.326	0.08			
Blood loss, mL						
≤300	1					
>300	1.097	0.565–2.131	0.784			
Surgical procedure						
PPPD	1					
Whipple	1.211	0.498–2.943	0.673			
Vascular resection						
No	1					
Yes	3.995	0.871–18.331	0.075			
Pancreatic texture						
Soft	1					
Firm	0.257	0.119–0.557	**0.001**	0.881	0.311–2.495	0.811
Duct diameter, mm						
≤3	1					
>3	0.248	0.128–0.482	**<0.001**	0.333	0.139–0.796	**0.013**
High-risk pathology						
Yes	1					
No	0.154	0.077–0.311	**<0.001**	0.277	0.106–0.727	**0.009**

BMI, body mass index; ASA, American Society Anesthesiology; PPPD, pylorus-preserving pancreaticoduodenectomy; OR, odds ratio; 95% CI, 95% confidence interval.
